# Identification, Quantification, and Antioxidant Evaluation of Phenolic Compounds from Colored *Opuntia ficus-indica* (L.) Roots Using UHPLC-DAD-ESI-MS/MS

**DOI:** 10.3390/antiox14081023

**Published:** 2025-08-21

**Authors:** Elias Benramdane, Ahmad Mustafa, Nadia Chougui, Nawal Makhloufi, Abderezak Tamendjari, Cassamo U. Mussagy

**Affiliations:** 1 Laboratoire de Biochimie Appliquée, Faculté des Sciences de la Nature et de la Vie, Université de Bejaia, Bejaia 06000, Algeria; 2 Faculty of Engineering, October University for Modern Sciences and Arts (MSA), Giza 12451, Egypt; 3 Département des Sciences Alimentaires, Faculté des Sciences de la Nature et de la Vie, Université de Bejaia, Bejaia 06000, Algeria; 4 Laboratorio de Desarrollo de Bioprocesos Sostenibles (Labisost), Escuela de Agronomía, Facultad de Ciencias Agronómicas y de los Alimentos, Pontificia Universidad Católica de Valparaíso, Casilla 4-D, Quillota 2260000, Chile

**Keywords:** *O. ficus-indica*, root extract, UHPLC-DAD-ESI/MS, antioxidant activity, sustainable cosmetics, antioxidants

## Abstract

This study investigates the phenolic composition and antioxidant potential of root extracts from three *Opuntia ficus-indica* varieties (green, red, and orange) using ultra-high-performance liquid chromatography coupled with diode array detection and electrospray ionization–tandem mass spectrometry (UHPLC-DAD-ESI-MS/MS). Phenolic compounds were extracted with a hydromethanolic solvent and quantified by spectrophotometric assays, while antioxidant activity was assessed through DPPH, ABTS, iron III reduction, hydroxyl radical, and nitric oxide scavenging methods. A total of 26 compounds were identified, including piscidic acid, epicatechin-3-*O*-gallate, and isovitexin, with several phenolics newly reported for *O. ficus-indica* roots. The green and red varieties showed the highest phenolic contents (up to 147.82 mg/g extract) and strong antioxidant capacity, particularly in ABTS (IC_50_ = 29.38 μg/mL) and hydroxyl radical inhibition (>90%). Relative Antioxidant Capacity Index (RACI) analysis confirmed a consistent correlation between phenolic/flavonoid content and antioxidant efficacy. These findings highlight the analytical relevance of UHPLC-DAD-ESI-MS/MS for profiling underutilized plant matrices and support the potential use of *O. ficus-indica* root extracts as natural sources of bioactive compounds for pharmaceutical and biomedical applications.

## 1. Introduction

The global shift towards a circular economy has significantly emphasized the valorization of agricultural side-streams [1]. Each year, large amounts of plant biomass, including roots, stems, and peels, are discarded, posing a substantial environmental challenge [2]. Transforming this biomass into value-added products is a key aspect of sustainable innovation. In this context, the root system of *O. ficus*-*indica* (prickly pear cactus), a plant renowned for its resilience and the commercial value of its fruit and cladodes, remains an underutilized resource. Despite its extensive root network, which is crucial for survival in arid ecosystems, this biomass is often treated as agricultural waste [3].

This underutilization starkly contrasts with the increasing consumer and industrial demand for natural, effective, and sustainably sourced ingredients, especially in the pharmaceutical and cosmetic sectors [4]. Bioactive compounds from plants, particularly those with antioxidant properties, are highly valued for their ability to mitigate oxidative stress, a key factor in cellular aging and various chronic diseases [5]. While other parts of the *O. ficus*-*indica* plant are well-documented sources of these phytochemicals, its roots have been largely unexplored scientifically. Preliminary studies, however, suggest they may possess notable biological activities, hinting at a hidden reservoir of valuable compounds [6,7].

This study was thus designed to conduct the first systematic investigation into the chemical composition and antioxidant potential of roots from three colored varieties of *O. ficus*-*indica* (green, red, and orange) from north Algeria. We hypothesized that these roots contain a diverse array of phenolic compounds responsible for significant antioxidant activity. To test this, we employed advanced analytical techniques, including ultra-high-performance liquid chromatography coupled with mass spectrometry (UHPLC-DAD-ESI-MS/MS), to create a comprehensive phytochemical profile. The antioxidant capacity was then rigorously evaluated using a series of in vitro assays to assess multiple mechanisms of action.

Finally, to bridge the gap between biochemical profile and practical application, we explored the feasibility of incorporating these bioactive-rich extracts into functional handmade soap formulations. This research not only highlights the untapped biochemical value of *O. ficus*-*indica* roots but also demonstrates a practical pathway for converting agricultural waste into a high-value ingredient for natural cosmetics. The findings contribute directly to the principles of a circular bioeconomy, offering a sustainable model for resource management in arid and semi-arid regions.

## 2. Materials and Methods

### 2.1. Chemicals

All solvents utilized for chemical analysis, including methanol and acetonitrile, were of high-purity HPLC grade (≥99.8%) and were procured from Fisher Scientific (Pittsburgh, PA, USA). The reagents for antioxidant assessments, such as 2,2-diphenyl-1-picrylhydrazyl (DPPH•), 2,2′-azino-bis(3-ethylbenzothiazoline-6-sulfonic acid) diammonium salt (ABTS), and ascorbic acid (≥99.5%), were of analytical grade and purchased from Sigma-Aldrich (Merck, Darmstadt, Germany).

### 2.2. Sampling of Roots

**Figure 1:**
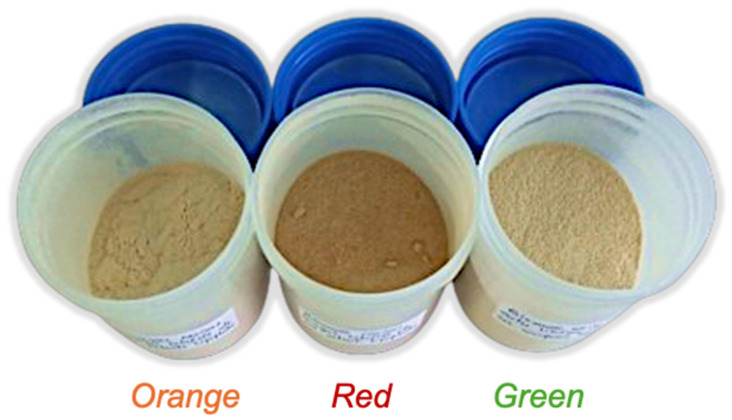
Ground powdered roots of three varieties—green, orange, and red—of *O. ficus-indica* from north Algeria.

Roots from three varieties of *O. ficus-indica* cactus (orange, green, and red) were collected from two distinct semi-arid regions in northern Algeria. The orange variety was harvested from the El-Kseur region, while the green and red varieties were sourced from the At-Wasif province. After collection, the roots were transported to the laboratory, where they were meticulously washed with distilled water to eliminate soil and other impurities. The cleaned roots were then cut into small cubes, freeze-dried using an LSCBasic Christ freez-dryer (Martin Christ Gefriertrocknungsanlagen GmbH, Osterode am Harz, Germany) to best preserve their phytochemical composition, and subsequently ground into a fine powder. This final biomass was stored in sealed containers under dry conditions prior to extraction (Figure 1).

### 2.3. Solid–Liquid Extraction of Phenolics Compounds

Phenolic compounds were obtained from the root powder using a solid–liquid extraction technique. For each cactus variety, one gram of powder was macerated in 100 mL of a methanol/water solution (1:1, *v*/*v*). This mixture was continuously stirred at 900 rpm for 24 h in light-protected flasks to prevent the photodegradation of sensitive compounds [8]. Following the extraction period, the mixture was centrifuged to separate the solid plant material. The liquid supernatant was filtered through a sintered glass Büchner funnel. Finally, the solvent was removed under low pressure using a rotary evaporator at 40 °C (SCILOGEX SCI100-S, Scilogex, Xingang, China), and the resulting concentrate was freeze-dried into a fine powder, which was used for all subsequent analyses.

### 2.4. Determination of Total Phenolic and Flavonoid Compounds

The total phenolic content (TPC) was quantified using the Folin–Ciocalteu spectrophotometric method in a 96-well microplate format. An aliquot of the extract was mixed with Folin–Ciocalteu reagent and a sodium carbonate solution, then incubated in the dark for one hour before its absorbance was measured at 750 nm [9]. The TPC was determined using a gallic acid standard curve and expressed as mg of gallic acid equivalents per 100 g of powdered roots (mg GAE/100 g pr). The total flavonoid content (TFC) was similarly assessed by mixing the extract with a 2% aluminum chloride solution. After a 15 min incubation at room temperature, the absorbance was read at 430 nm [10]. The TFC was calculated from a quercetin standard curve and expressed as mg of quercetin equivalents per 100 g of powdered roots (mg QE/100 g pr).

### 2.5. UHPLC-DAD-ESI/MS^n^ Investigation

The extracts of cactus roots were dissolved in a binary mixture of methanol and water (1:1, *v*/*v*) at a concentration of 10 mg mL^−1^. The solution was filtered through PTFE filters with 0.2 µm pore diameter. An amount of 10 µL of the filtered solution was injected into the UHPLC system consisting of an Accela 600 LC pump, an Accela autosampler, and an Accela 80 Hz photo diode array detector (DAD) (Thermo Fisher Scientific, San Jose, CA, USA). The separation of the components was carried out using a Hypersil Gold RP C18 column (100 × 2.1 mm; 1.9 µm particle size) preceded by a C18 pre-column (2.1 mm i.d.) supplied by Thermo Fisher Scientific (San Jose, CA, USA); both columns were kept at 45 °C. A gradient elution program was used with the mobile phase consisting of water/acetonitrile (99:1, *v*/*v*) (A) and acetonitrile (B), both containing 0.1% (*v*/*v*) formic acid. The elution program involved a flow rate of 0.45 mL/min and gradient elution from 1% B kept from 0 to 3 min, 1–31% B from 3 to 30 min, 31–100% B from 30 to 32 min, and 100–1% B from 32 to 36 min, followed by a re-equilibration step with 1% B from 36 to 40 min. Chromatograms were recorded at 235, 280, and 370 nm and UV-Vis spectra from 210 to 600 nm. The UHPLC system was coupled to a LCQ Fleet Ion Trap (FIT) mass spectrometer (Thermo Finnigan, San Jose, CA, USA) equipped with an Electro-Spray Ionization (ESI) source and operated under negative ionization mode with a spray voltage of 5 kV and capillary temperature of 320 °C. CID-MSn experiments were carried out on mass-selected precursor ions in the range of *m*/*z* 100–2000. The data were acquired using the Xcalibur^®^ data system (Thermo Finnigan, San Jose, CA, USA).

Raw mass spectrometry data were converted to the Mascot Generic Format (.mgf) using the ProteoWizard (version 3.0.2) software suite, with vendor-specific peak picking enabled to centroid the spectra [11]. For comprehensive de novo metabolite identification, the resulting files were compared to the results found in previous studies but also processed with the SIRIUS-GUI (version 5.8.6) platform in order to confirm the identification. This platform integrates a cascade of tools to achieve multi-layered annotation. Initially, it predicts molecular formulas from isotopic patterns and fragmentation trees, which are then refined at a global dataset level by the ZODIAC module. Subsequently, the CSI:FingerID tool predicts molecular fingerprints to query against structural databases, with the COSMIC module providing a confidence score for each structure–spectrum match. Finally, the CANOPUS module uses a deep learning approach to assign all annotated compounds to chemical ontology classes. This integrated workflow provides a robust, high-confidence characterization of detected metabolites, progressing from molecular formula to structural hypotheses and ultimately chemical classification [12].

For the estimation of individual compound concentration, we utilized the relative peak area percentage for each identified compound from the UHPLC chromatograms, obtained using Thermo Xcalibur (version 2.2) software. This surface peak percentage represents the contribution of each compound to the total chromatographic area of all identified phenolics. By correlating this relative percentage with the absolute TPC value (in mg/g of extract) from Table 1, we calculated the estimated concentration of each individual compound presented in Table 2. The calculation was performed as follows for each compound.

$$Concentration\left(µg/\mathrm{mg}\mathrm{extract}\right)=\left(\frac{Total\;Phenolic\;Content\times Peak\;area\%\;}{100}\right)\times 1000$$

To ensure the reliability and precision of the results, each extract was analyzed in two independent replicates. The entire analytical procedure was then repeated three times for each sample, resulting in a total of six measurements (*n* = 6) for each extract.

### 2.6. Antioxidant Activities

Antioxidant activities were assayed using different tests. All tests were performed in triplicate, and each experiment was repeated three times (*n* = 9), with an extract stock solution of 10 mg/mL for each assay, the details are described below.

The DPPH• (2,2-diphenyl-1-picrylhydrazyl) radical scavenging assay was used to measure the hydrogen-donating potential of the root extracts. Various concentrations of each extract were added to a stable methanolic solution of the purple-colored DPPH• radical. After a 30 min incubation period in the dark, the ability of the extract’s antioxidants to reduce the radical was quantified by the decrease in color intensity, measured as a change in absorbance at 520 nm [37].

An ABTS assay determined the extracts’ capacity for radical quenching through electron transfer. The blue-green ABTS•^+^ radical cation was first generated by reacting an aqueous solution of ABTS with potassium persulfate. This radical solution was then diluted to a standard absorbance and mixed with the extracts. The reduction in the radical’s color, indicating antioxidant activity, was measured spectrophotometrically at 750 nm after 30 min of reaction time [38].

The iron III reduction assay was performed to assess the ability of the extracts to reduce ferric iron (Fe^3+^) to its ferrous form (Fe^2+^). Each extract was mixed with a phosphate buffer and potassium ferricyanide solution and incubated at 50 °C. Following this, trichloroacetic acid was added to stop the reaction. The concentration of the resulting ferrous iron, which produces a colored complex, was determined by reading the absorbance at 700 nm [39].

The capacity of the extracts to scavenge nitric oxide (NO•) radicals was evaluated using sodium nitroprusside as a generator. Extracts were incubated with the nitroprusside solution under a light source to induce the release of NO•. After the incubation period, the amount of remaining NO• was quantified using the Griess reagent, which detects the presence of nitrite formed from the radical, with the final absorbance measured spectrophotometrically [40].

The scavenging activity against the highly reactive hydroxyl radical (•OH) was determined using a Fenton reaction system. Hydroxyl radicals were generated by mixing ferrous sulfate (FeSO_4_) and hydrogen peroxide (H_2_O_2_). The protective effect of the extracts was measured by their ability to inhibit the hydroxylation of sodium salicylate. After a one-hour incubation, the absorbance of the reaction mixture was read at 560 nm to quantify the degree of scavenging [41].

### 2.7. Relative Antioxidant Capacity Index (RACI)

To obtain a complete and detailed picture of the ranking of the antioxidant potentials of the studied *O. ficus-indica* root extracts, relative antioxidant capacity scores were calculated by integrating the values of the results generated from different tests [42]. A score was calculated for each antioxidant test (IC_50_ and percentage of activity) for each variety studied. The RACI is the standard average value of the scores transformed from the initial data generated with different methods, presented as an overall score, and its value varies from −1.5 to 1.5 in this study. The *RACI* was calculated using the following formula:

$$RACI\;=\;\frac{Sample\;value-Sample\;mean}{Standard\;Deviation}$$

### 2.8. Preparation of Extract-Enriched Soap Formulations

A commercially available neutral soap bar was sourced from a local supplier to serve as the base material. For each formulation, 10 g of the soap base was weighed and slightly moistened with 1–2 mL of deionized water to aid in melting. The soap was then transferred to a glass beaker and melted on a hot plate set to 65–70 °C, with continuous gentle stirring until fully liquefied. Once melted, *O. ficus-indica* root extracts were added to achieve three final concentrations, 3.33 mg/g (33.3 mg extract per 10 g soap; Soap 1), 5.00 mg/g (50 mg extract per 10 g soap; Soap 2), and 10.00 mg/g (100 mg extract per 10 g soap; Soap 3), based on the extract-to-soap base ratio (*w*/*w*). Each concentration was prepared separately using root extracts from three different *O. ficus-indica* varieties—orange, red, and green—resulting in nine distinct formulations. After thorough mixing, the enriched soap mixtures were poured into silicone molds and allowed to solidify at room temperature for 24 h. The solidified soaps were then demolded and cut into uniform cubes for further analysis. The antioxidant activity of the enriched soaps was assessed using DPPH and ABTS radical scavenging assays, as described in Section 2.6.

### 2.9. Color Analysis

The color parameters of the soap samples were determined using the CIELab color space system, which provides quantitative values for lightness (L*), red-green chromaticity (a*), and yellow-blue chromaticity (b*). A colorimeter (LS173, Shenzhen, China) was employed for the measurements. Prior to analysis, the instrument was calibrated using the standard white tile provided by the manufacturer. Measurements were carried out under standard conditions using D65 as the illuminant and a 10° standard observer angle. Each soap sample was analyzed in triplicate at different surface points, and results were expressed as mean values. Additionally, the total color difference (ΔE*ab) relative to the control sample (soap without *O. ficus-indica* extract) was directly provided by the equipment’s internal software, allowing immediate comparison of color variations among the different formulations.

### 2.10. Statistics

The graphs were plotted with the use of GraphPad Prism 8.0.1 software (San Diego, CA, USA). Triplicate-performed tests are averaged and presented as means ± SD. Variance analyses were performed by Tukey’s HSD post hoc ANOVA test; the two analyses were performed using Statistica 7.1 (Statsoft^®^, Tulsa, OK, USA). In all cases, 0.05 was fixed as a significative threshold.

## 3. Results and Discussion

### 3.1. Total Phenolic and Flavonoid Content

The levels of total phenolic and flavonoid compounds in the roots of three varieties of *O. ficus-indica*, expressed in grams of extract, are presented in Table 1.

As depicted in Table 1, the extract of the green variety contains the highest concentration of total phenolic compounds, with a value of 147.82 ± 10.33 mg/g, followed by those of the red (120.29 ± 3.51 mg/g) and orange (100.83 ± 6.11 mg/g) varieties (*p* < 0.05). These concentrations are two to three times higher than those of the roots of the thornless cultivar of *O. ficus-indica* reported in a Tunisian study on the gastroprotective effect of the roots of this species (57.56 ± 0.51 mg/g) [6]. In addition, a considerable variance is observed when comparing them to the contents of the fruit (9.94 ± 0.01–16.51 ± 0.01 mg/g) [43]. This could be explained by the morphological differences between these botanical parts, as well as their exposure to different biological phenomena. The yield per 100 g of root powder shows much higher contents in the different varieties, the richest of which corresponds to the red variety, with a value of 2944.56 ± 6.31 mg/100 g pr, followed by the green variety (2920.19 ± 42.21 mg/100 g pr), where the difference is not significant (*p* > 0.05), unlike for the orange variety (1392.63 ± 57.95 mg/100 g pr). These results agree with those reported for young and adult cladodes of the same species (1890 and 1480 mg/100 g pr) [44]. However, values 1.5 to 2 times higher were observed in another study on cladodes dried at 45 °C with variable air flows (4100 mg/100 g pr) [45]. Indeed, the same values are 2.5 to 4 times higher than the contents recorded in seeds of the same species (504.8 mg/100 g pr) [46].

The levels of flavonoids represent 82 to 88% of the total phenolic compounds measured in the roots of the three varieties studied, both for the hydromethanolic extracts and for the root powders (Table 1). Indeed, the highest level was found in the red variety (87.8%). Our results are slightly higher than those already reported in [47] for the cladodes of *O. ficus-indica*. Among the three varieties studied, the highest concentration per gram of extract is found in the green variety, with 125.65 ± 6.33 mg/g, followed by that of the red variety (105.60 ± 3.88 mg/g) and finally that of the orange variety (82.33 ± 7.18 mg/g)—the same order as for the total phenolic compound contents (*p* < 0.05). These concentrations are 3 to 5 times higher than those of the roots of the inerm cultivar (23.5 ± 0.67 mg/g) from a Tunisian variety [6] but are closer to the flavonoid content of the cladodes (71.02 ± 0.76 mg/g) [47]. The yield of flavonoids, expressed in milligrams per 100 g of root powder, is higher in the roots of the red variety. Indeed, as was the case for total phenolic compounds, this variety is shown to be the richest, with a content of 2591.18 ± 5.29 mg/100 g pr; this not too far from the value for the green variety. The lowest content was found in the orange variety, which is approximately 2 times lower (1141.96 ± 33.3 mg/100 g pr). The differences between the three varieties are significant at *p* < 0.05. The content of phenolics may vary depending on several factors, including the growing conditions of wild *O. ficus*-indica cladodes [48].

### 3.2. Identification of Phenolic Compounds

The phytochemical screening of *O. ficus-indica* roots belonging to three varieties was performed using UHPLC-ESI-MSn with two levels of peak fragmentation. About fifty mass spectra (MS) were analyzed and compared for each variety. This in-depth analysis of the composition of the hydromethanolic root extracts allowed the identification of 26 phytochemicals, which were mainly flavonols, flavones, and glycosylated flavonols, hydroxybenzoic acids and derivatives, and organic acids (Table 2). The identification criteria used in this study were the elution time and order as well as the fragmentation, at two levels of the compounds, by comparing them with those already reported in the literature.

#### 3.2.1. Organic Acids

Two organic acids were detected in our extracts (compounds **1** and **2**) (Table 2). By comparison of their mass spectra, which are 133 and 191, respectively, these organic metabolites are already identified in the cladodes and fruit of the *O. ficus-indica* species [13,14].

#### 3.2.2. Phenylpyruvic Acids

Compounds **3** and **4** are, respectively, identified as two phenylpyruvic acids, piscidic and eucomic, according to their M-H, which are 255 and 239, respectively, as well as their elution time and order (Table 2). These two compounds are major phenolic acids found in different botanical parts of the cactus, notably the fruit peels and cladodes [14,49,50]. The red-variety extract contained the highest estimated content of piscidic acid at approximately ~1.91 µg/mg. While this compound is a well-known marker for the *Opuntia* genus, it has primarily been quantified in cladodes. Notably, a study by Blando et al. [49] on Italian *O. ficus-indica* cladodes reported concentrations ranging from 1.98 to 3.28 mg/g dry weight, placing the content in our red root variety within the same order of magnitude as that of mature cladodes and highlighting its systemic importance in the plant. The highest concentration of eucomic acid was in the green variety (~2.36 µg/mg). For comparison, Blando et al. [49] found substantially higher levels in immature cladodes (13.5 mg/g) but a comparable level in mature cladodes (1.6 mg/g). This suggests that the eucomic acid content in roots is similar to that of aged, mature cladode tissues.

#### 3.2.3. Glycosylated Flavonols

**Figure 2:**
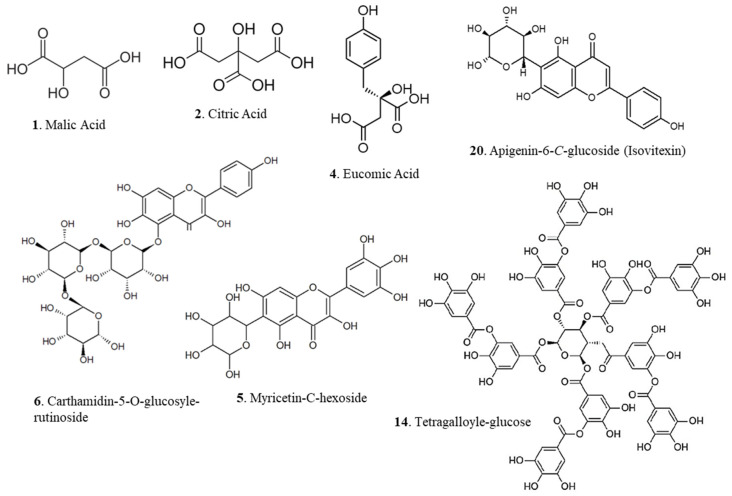
Major phenolic compounds identified in the hydromethanolic root extracts of three varieties of *O. ficus-indica*.

Glycosylated flavonoids are the major constituents of the hydromethanolic extracts of *O. ficus-indica* roots. In the full-scan mass spectra, most ions were selected to undergo dissociation by CID (Collision-Induced Dissociation) in the MS/MS spectrum. Although flavonoids can be ionized under both positive and negative modes, the most intense signals are obtained with the negative mode. The glycosylated flavonols presented below are identified for the first time in the *O. ficus-indica* species. Compound **5** gave a base peak of M-H = 479 and an initial fragmentation of *m*/*z* 359; considering the spectra reported by [15] it was identified as an isomer of myricetin-*C*-hexoside (Figure 2, Table 2). The spectra of compound **6** exhibited a base peak of M-H = 757 and an MS/MS fragmentation of *m*/*z* 595, the latter being the loss of most of the adducted hexoside. This fragmentation is specific to carthamidin-5-*O*-glucosyl-rutinoside (Figure 2), with the fragment *m*/*z* 287 representing the aglycone form of the molecule [16]. Compound **7** has already been identified in its aglycone form in the roots of *O. ficus-indica* in South Korea. As the only molecule identified in the roots of this species, this antioxidant molecule with anti-diabetic effects is identified in its triglycosylated form in this study, with a base peak of M-H = 743. A series of fragments are subsequently highlighted up to *m*/*z* 288, representing the aglycone form of this dihydroflavonol derivative, otherwise known as dihydrokaempferol [17]. The fragmentation after the ionization of compound **12** corresponds exactly to that reported by Mascherpa et al. [21], showing a base peak of M-H = 611 corresponding to kaempferol *O*-diglucoside; a first fragmentation at *m*/*z* 449 corresponding to the loss of half of the adducted disaccharide, as well as another ion *m*/*z* 287 specific to kaempferol in aglycone form. The green variety also showed the highest content of kaempferol-*O*-diglucoside (~1.77 µg/mg). This is a particularly interesting result when compared to the aerial parts of the plant. While specific data for the diglucoside are scarce, its aglycone, kaempferol, and its common rutinoside form are abundant. Guevara-Figueroa et al. [48] reported kaempferol-3-*O*-rutinoside in Mexican cladodes at levels up to 1465 µg/g, while Ammar et al. [51] found kaempferol-3-*O*-rutinoside at 6.46 mg/100g in Tunisian flowers. The vastly different concentrations and glycosidic forms between the roots and aerial parts underscore a specialized metabolism within the root tissue.

Compound **13** is also considered a derivative of kaempferol, and this triglycosylated molecule showed a deprotonated ion of M-H = 787, a first fragmentation at *m*/*z* 625 and *m*/*z* 463 corresponding to the loss of sugar moieties (glucose/galactose), and finally a hydroxylated aglycone form with an *m*/*z* of 301 representing hydroxy-kaempferol [16]. Compound **16** displayed an M-H = 593, and in the MS2 spectrum there were two fragments at *m*/*z* 285 and *m*/*z* 284 that suggested that the hexose and deoxyhexose are linked to the same position in the aglycone (kaempferol). The ion produced at *m*/*z* 447 is attributed to the neutral loss of deoxyhexose [25].

The last glycosylated derivative is identified at RT = 28.02 min and corresponds to the elution of quercetin-3-*O*-(6″-acetyl) hexoside (compound **21**). This molecule exhibited an M-H = 505, and after MS2 analysis, the acetyl residue is released with a loss of 42 units to give a fragment at *m*/*z* 463. Subsequently, the loss of the hexosyl part is marked by the appearance of the specific *m*/*z* 301 ion for quercetin aglycone [32,33]. Similarly, the green root variety contained quercetin-3-*O*-(6″-acetyl) hexoside at ~1.74 µg/mg. This again contrasts with the cladodes and flowers, where other forms of quercetin dominate. For instance, the common quercetin-3-*O*-rutinoside (rutin) was found at concentrations up to 261.7 µg/g in cladodes [48] and an impressive 390.27 mg/100g in Tunisian flowers [51]. The presence of a different, acetylated quercetin derivative in the roots highlights a unique enzymatic machinery not seen in the rest of the plant.

#### 3.2.4. Flavanols and Flavones

Compound **11** is the typical molecule of Chinese green tea. A flavanol class compound already identified in the young cladodes of *O. ficus-indica* [52], epicatechin 3-*O*-gallate (EGCG), is detected in this study with M-H = 441 and a major fragment of *m*/*z* 289, which corresponds to epicatechin, a molecule with anti-diabetic (type II) and triglyceride-lowering effects in humans [53,54,55]. Peak 20 showed a UV spectrum typical of flavone *C*-glucosides. MS2 analysis allowed the identification of a relatively intense ion at *m*/*z* 311 [M-H-120] that corresponded to the loss of a part of the glucose attached to carbon 6 of apigenin, according to Kang et al. [31]. The representative ion of the aglycone form of this molecule is at *m*/*z* 269; therefore, compound **20** is identified as apigenin-6-*C*-glucoside, otherwise known as isovitexin (Figure 2). Compound **22**, with M-H = 329, produced fragments at the same value (*m*/*z* 329) and other major ions at *m*/*z* 229, *m*/*z* 211, and *m*/*z* 183 [34]; the characteristics of this fragmentation suggest the flavone tricin. The most striking differences among the varieties were found in their flavonoid profiles, which likely govern their distinct bioactivities. The red variety was exceptionally rich in apigenin-6-*C*-glucoside (isovitexin), with an estimated content of ~4.47 µg/mg. While Aruwa et al. [56] have qualitatively identified isovitexin in the cladodes of South African *O. ficus-indica*, it has never been quantified as a major component. Its high concentration in the roots is therefore a novel and important finding, positioning the red variety as a unique source for this specific bioactive flavonoid. In sharp contrast, the green variety extract was distinguished by having the highest estimated content of epicatechin-3-*O*-gallate (EGCG) (~1.79 µg/mg). EGCG is a powerful antioxidant, and while our value is modest, it is significant. Previous work on Moroccan cladodes by Boutakiout et al. [52] reported a substantially higher EGCG content of 13.18 µg/g. This indicates that while the roots are a source of EGCG, the cladodes are a much richer reservoir.

#### 3.2.5. Phenolic Acid Derivatives

Compound **8** (Table 2) showed an M-H = 535, and the *m*/*z* 329 fragmentation is formed due to the loss of the sinapoyl residue M-H-206. The second fragment *m*/*z* 367 gave rise to the deprotonated ion *m*/*z* 205 after the loss of the glucose part (162 units) M-H-168-205. Therefore, this compound is identified as 6″-*O*-sinapoyl gardoside [18]. Compound **10** is characterized as a guaiacyl-glyceryl derivative of ferulic acid due to the detected ion at *m*/*z* 613, which gave rise to the base peak at *m*/*z* 569, indicating the loss of a molecule of CO_2_. The *m*/*z* 196 fragment is attributed to the guaiacyl-glyceryl part [19].

Interestingly, while we did not detect free ferulic acid, we found a high concentration of a ferulic acid guaiacylglyceryl derivative in the green variety (~1.84 µg/mg). This is significant because free ferulic acid is one of the most abundant phenolic acids in cladodes, with concentrations reported to be as high as 347.7 µg/g [48]. The presence of its complex derivative in the roots suggests a distinct metabolic pathway where this common precursor is utilized for synthesizing more complex structural or defensive molecules in the subterranean parts of the plant. Compounds **8** and **10** are newly discovered in this species. The last compound identified in this class is number 26, eluted at RT = 32.65 min; its M-H = 537 lost a value of 162 units attributed to the glucose part attached to the compound after the first fragmentation [13]. The fragmentation of the *m*/*z* 375 ion (sugar-free molecule) gave rise to an ion at *m*/*z* 327, indicating the loss of a molecule of H_2_O and a methanal group (CH_2_O), as well as an ion at *m*/*z* 179 related to guaiacyl. Therefore, compound **26** is considered to be guaiacyl 8-*O*-4-guaiacyl-hexoside (glucoside), based on the spectra reported [13].

#### 3.2.6. Flavonoid Derivatives

Compound **9** (Table 2) gave an ion M-H = 569, and a fragmentation at *m*/*z* 345 was detected, corresponding to the aglycone form of Spinacetin. A loss of 224 units representing the sinapic acid moiety was observed, confirming the identification of this compound as sinapoyl spinacetin [19]. The fragmentation of compound **19** was assigned to that of procyanidin B1, according to [30]. Indeed, the MS and MS2 spectra of this compound gave an M-H = 577 relative to a dimer of catechin/epicatechin, as well as an ion at *m*/*z* 289 corresponding to the monomer of the same molecule. Finally, peak 23 is assigned to isovitexin-3-hydroxy-3-methylglutaryl, based on its M-H = 575 and fragmentation, which mainly gave an ion at *m*/*z* 431, indicating a loss of 144 units corresponding to the 3-hydroxy-3-methylglutaric acid moiety [35]. Compounds **9** and **23** (Table 2) are identified for the first time in *O. ficus-indica*.

#### 3.2.7. Other Identified Compounds

The compounds related to molecules **14**, **15**, **18**, **24**, and **25** (Table 2) are also identified for the first time in our species. With a mass of M-H = 787, compound **14** is characterized as tetragalloyl glucose according to Mämmelä et al. [23](Figure 2), due to the appearance of the major fragment *m*/*z* 635 indicating a loss of 152 units attributed to the galloyl part [22], as well as another fragment *m*/*z* 331 assigned to the remaining galloyl glucose part [57]. With the green variety extract showing the highest estimated content at approximately ~2.15 µg/mg, this finding is particularly significant, as according to the available literature, tetragalloyl-glucose, a complex hydrolysable tannin, has not been previously reported in the more extensively studied parts of the plant, such as the cladodes, flowers, or peels. While the basic building block gallic acid is a well-known constituent of cladodes, the discovery of this highly galloylated glucose derivative in the root’s points to a specialized metabolic pathway capable of producing high-molecular-weight tannins [58]. Hydrolysable tannins are known for their potent biological activities, including strong protein-binding and enzyme-inhibiting properties, which differ significantly from simple phenolic acids [59]. Therefore, this discovery not only adds a new class of compounds to the known phytochemistry of *O. ficus-indica* roots but also significantly enhances their potential value as a source of high-potency natural ingredients.

The mass spectra of compounds **15**, **18**, and **25** are similar to those reported by other authors, who confirmed their identifications as the three acids, poricoic, yunnanonic, and placodioic, respectively [24,28,36]. Compound **24** is identified as a hydroxy fatty acid, its mass spectrum is similar to that reported by Kang et al. [31], who characterized a hydromethanolic extract of a tropical herbaceous plant in a study similar to the present one. The presence of hydroxyl groups makes the molecule more polar [60], especially as the retention times are similar between the two studies. The MS2 analysis of this compound shows successive losses of water molecules and aliphatic segments of the identified fatty acid, trihydroxy-octadecadienoic acid. Finally, another compound belonging to the dihydrochalcones, phloretin-2′-xyloside (**17**), indicated a deprotonated ion at *m*/*z* 567 and a fragment at *m*/*z* 273, the latter clearly being due to the loss of 132 units corresponding to the xyloyle part (sugar adduct) [27].

### 3.3. Antioxidant Evaluation

The results of antioxidant activities conducted with five different tests are summarized in Table 3.

Table 3 shows the effective concentrations that reduce 50% of the radical and non-radical reactive species (IC_50_, EC_50_). The first observation reveals that the hydromethanolic extracts of the studied *O. ficus-indica* roots are more effective against the synthetic radical ABTS•^+^ and at reducing ferric iron (Fe^3+^) at concentrations below 100 μg/mL for all three varieties. Secondly, relatively higher concentrations are observed against the two reactive radical species, namely the hydroxyl radical (•OH) and the nitric oxide, also known as nitrogen oxide (NO•), ranging from 120 to 190 μg/mL. Subsequently, a slight increase in concentration was necessary for the reduction of phosphomolybdenum, with doses ranging from 250 to 270 μg/mL. Finally, the lowest effectiveness is recorded in the trapping of the synthetic radical DPPH•, of which the highest IC_50_ values were between 700 and 850 μg/mL.

#### 3.3.1. DPPH Radical

**Figure 3:**
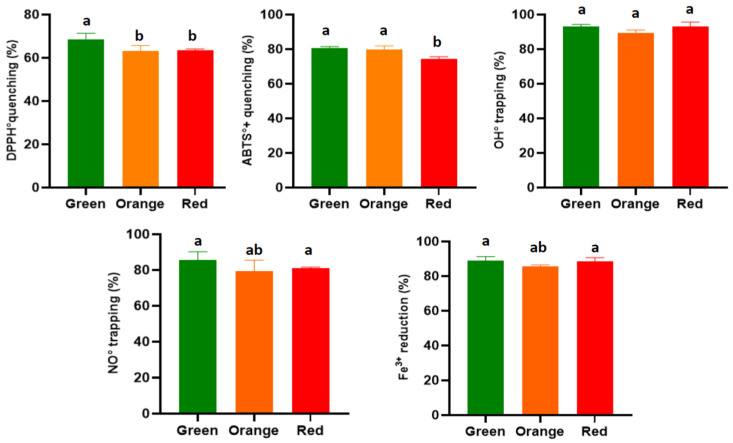
Histograms of the percentages of the anti-free-radical activities of phenolic extracts from the roots of 3 varieties of *O. ficus-indica*. The difference in the letters in the histograms indicate a significant difference between varieties (*p* < 0.05).

The anti-radical activity through hydrogen transfer (DPPH•-DPPH_2_^+^) gave a maximum inhibition percentage of 68.65 ± 2.73% in the extract from the green variety, which was significantly higher than the extracts from the orange and red varieties, which showed lower activities of 63.00 ± 2.68% and 63.60 ± 0.49%, respectively (Figure 3). These rates are lower than those obtained by Alimi et al. [6] for the hydromethanolic fraction of thornless *O. ficus-indica* roots (78.01 ± 4.11%); higher than the results reported by Avila-Nava et al. [61] for the non-hydrolyzed fraction of the hydromethanolic extract of cladodes of the same species, which indicated a 40% inhibition of this radical; and higher than the inhibition percentage of the ethanol extract from the same species from South Korea in a study conducted by Jeon et al. [62], which yielded a rate of 47.94 ± 0.13%. The IC_50_ values obtained in the present study showed the same trend as the results of previous studies. Similarly, the green variety proved to be more effective, with a value 695.70 ± 59.30 μg/mL, while the orange and red varieties came in a similar order, with values of 830.37 ± 72.33 and 852.29 ± 28.23 μg/mL, respectively. A significant difference was noted between the latter two varieties and the green variety (Table 3). The latter two concentrations are similar to those reported for the hydromethanolic extracts of the cladodes (867 ± 33 μg/mL) [61]. In contrast, the IC_50_ value obtained using the extract from the green variety was found to be twice as high as that reported for the cladode extract in a separate study [47]. The results we report in this study are much more relevant than those obtained for the fruit of *O. ficus-indica* in a South African study, where IC_50_ values ranging between 40 and 300 mg/mL were noted for methanolic extracts dried using two different methods [43]. Finally, the activities we report here are still lower than that of vitamin C, which is used as an antioxidant standard (5.87 ± 0.26 μg/mL).

#### 3.3.2. ABTS Radical

For this second synthetic radical, the root extracts were found to be more effective in reducing the ABTS•^+^ compared to its non-radical form by electron transfer. The inhibition rate reached 80.55 ± 0.91% with the extract from the green variety, while that of the orange variety was found to be in the same range, with a rate of 79.90 ± 1.96%; the extract from the red variety was significantly less effective, with an inhibition of 74.37 ± 1.31% (*p* < 0.05) (Figure 3). These rates are significantly higher than the activities of the hydro-methanolic extracts of the aerial part of this plant in different granulometric classes (where the inhibition rates range from 35 to 65%) [63], while they are lower than the results reported by Jorge et al. [64], who reported inhibitions greater than 90%. The best IC_50_ was recorded for the extract from the green variety (29.38 ± 1.21 μg/mL), which correlated with its high content of total phenolic compounds. The extracts from the orange and red varieties followed, with values of 31.01 ± 1.13 and 35.12 ± 0.95 μg/mL, respectively (Table 3). These results are significantly better than those reported by Petruk et al. [65] for the crude extract of the aerial part of this plant (IC_50_ = 520 ± 10 μg/mL). It is also noteworthy that our extracts, especially the extract from the green variety, exhibited activities four times weaker than that of TROLOX, which was used as the equivalent standard and had an IC_50_ of 6.70 ± 0.25 μg/mL. Based on the results of these two tests, it is noticeable that our extracts are more effective in reducing reactive species by electron transfer than by hydrogen transfer, a fact that has already been demonstrated for the extracts of this plant, with studies reporting significantly lower concentrations of ABTS•^+^ reducing activity compared to the stable DPPH• radical [53]. The same author reported an IC_50_ against ABTS•^+^ of 25.40 ± 0.90 μg/mL.

#### 3.3.3. Hydroxyl Radical OH

The in vitro results obtained in this study demonstrate that the hydromethanolic extracts of the roots of *O. ficus-indica* are highly effective in trapping this radical, shown by higher inhibition percentages compared to other antioxidant tests. A rate of 93.10 ± 2.50% was recorded with the extract from the red variety; the extract from the green variety had a similar rate (93.03 ± 1.23%), and that of the orange had a slightly lower percentage of 89.56 ± 1.50% (*p* > 0.05) (Figure 3). Therefore, we report for the first time, the effect of root extracts of this plant against the hydroxyl radical. These results are more relevant than those already reported by Lee et al. [66], who obtained inhibition rates that slightly exceeded 50%, whether by the deoxyribose technique or with ammonium thiocyanate. The IC_50_ values obtained against this radical ranged from 125 to 165 μg/mL, with the green variety exhibiting the lowest concentration (125.33 ± 2.66 μg/mL); the red variety had an IC_50_ value of 163.45 ± 5.55 μg/mL, which was slightly higher than that of the orange variety. Significant differences were noted between the varieties at *p* < 0.05 (Table 3). These results, particularly those for the green variety, are in perfect agreement with those already reported for the aqueous fraction of the pressed *O. ficus-indica* cladode extract, which had an IC_50_ equal to 98.24 ± 2.5 μg/mL in an *in vitro* study [67]. Furthermore, in another study on the same part of the plant, the IC_50_ obtained against this radical was 5600 ± 400 μg/mL [61], and, with a maximum inhibition percentage of less than 80%, this extract is significantly less effective than those used in this study.

#### 3.3.4. Nitric Oxide

Root extracts of *O. ficus-indica* tested in this study demonstrated inhibition rates of nitric oxide between 79 and 85%. The highest percentage (85.55 ± 4.66%) was recorded in the green variety (Figure 3). These results suggest root extracts are more effective than cactus seed extracts, based on the findings by Chaalal et al. [68], who reported inhibition rates not exceeding 35%. Furthermore, our results also indicate higher efficacy when compared to the results of another study reporting the anti-NO effect of *O. stricta* cladodes, a species very close to *O. ficus-indica* (50%) [69]. In an in vivo study, a strong inhibition of nitric oxide production of 77.2% by a flavonoid extract of cladodes was observed in the exudates of a laboratory rat inflammation model [70]. This value very close to the percentage found for the orange variety and the results for the other two varieties in this study.

The antioxidant activity varies depending on the type of extraction and the solvent used for this purpose. In an in vivo study, the authors observed a slight decrease in nitric oxide production in human chondrocytes at a concentration of 200 μg/mL [71]. Comparing this to the IC_50_ obtained in this study, it is clear that the cladode extract obtained by pressing is significantly weaker than our extracts, which showed 50% inhibition of this radical at a concentration of 123.82 ± 4.66 μg/mL for the green variety, followed by significantly higher values (*p* < 0.05) for the red and orange varieties, with values of 182.99 ± 4.56 and 189.30 ± 3.33 μg/mL, respectively (Table 3). Finally, these IC_50_ results also indicate a greater effect when compared to the inhibitory concentrations of peroxynitrite (ONOO^−^) (800 ± 30 μg/mL) obtained by Ref. [61] in an *in vitro* study.

#### 3.3.5. Reduction of Ferric Iron

**Figure 4:**
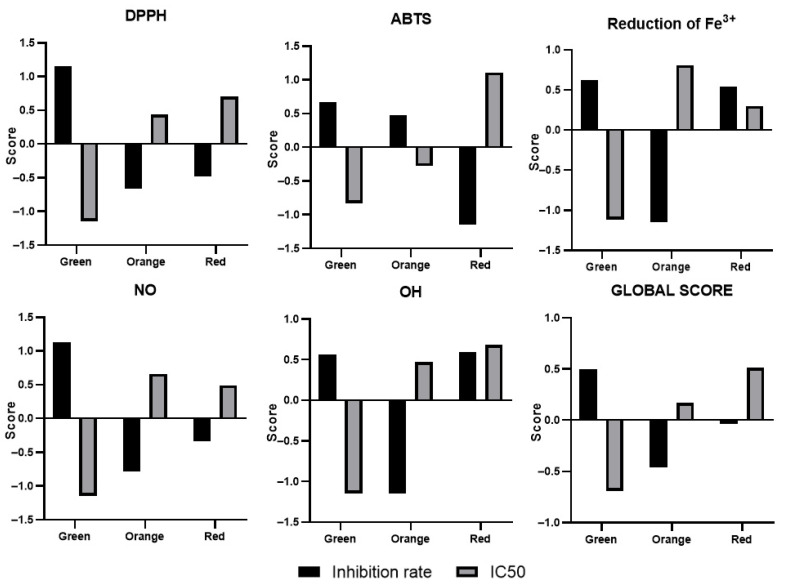
RACI scores calculated for each antioxidant activity of *O. ficus-indica* root extracts, as well as for the sum of all tests conducted (global score).

The reducing power of hydromethanolic extracts from the roots of *O. ficus-indica* revealed the highest percentages after the hydroxyl radical trapping test (Figure 4). These percentages were 88.8 ± 2.45% and 88.66 ± 1.99%, respectively, for the green and red varieties, which exhibited the highest values, followed closely by the orange variety, with a reduction rate of 85.66 ± 0.77%; the difference in this case was not significant. These results were higher than those obtained for the roots of inerm *O. ficus-indica*, which had a rate lower than 70% [6]. The IC_50_ values obtained using this test are shown in Table 3. The most effective concentration was noted in the green variety at 63.77 ± 2.33 μg/mL, followed by the red and orange varieties, with values of 66.87 ± 5.23 and 67.99 ± 1.77 μg/mL, respectively. These concentrations were twice as high as those obtained against the ABTS•^+^ radical but are very close to those for other antioxidant tests. Alimi et al. [6] obtained an IC_50_ of 300 μg/mL with the root extract of the inerm cactus in the FRAP test, a value five times higher than those reported in this study. For the aerial part, the ethyl acetate extract demonstrated an effective concentration of 125 ± 0.4 μg/mL, which is unlike the values obtained for ethanolic extract of the cladodes, as shown in the study conducted by Bakari et al. [72]. Other studies have reported average values between 62 and 87 μmol Fe (II)/g for the methanolic extract of the cladodes of the same species [56]. Finally, our extracts exhibited an efficacy close to that of the roots of *Inula racemosa*, which presented an IC_50_ of 2.72 ± 0.32 μM/μg of extract [73].

### 3.4. Relative Antioxidant Capacity Index—RACI

The calculation of the RACI (Relative Antioxidant Capacity Index) allowed us to see, for each antioxidant test, the varieties that have a strong potential against the above-described oxidizing elements, by giving a scores for the inhibition percentage, the EC_50_, and the IC_50_. This score varies from −1.5 to 1.5 for each activity in this study, with an overall score at the end describing the variety that presents the highest potential for all tests performed (Figure 4). For most antioxidant tests, the extract from the green variety gave the highest scores and stood out from the other extracts. This is due to the phenolic compound content of the extract, as well as the quality of the molecules present. Moreover, if positive and negative scores are obtained for the IC_50_ and the activity percentage, respectively, this could be explained by the fact that the extract contains high-quality molecules but at low concentrations.

Although a correlation test could not be established between the antioxidant activity and phenolic compound content results, after the calculation of RACI, this correlation can indeed be established, which is why the overall score is calculated. However, it is not possible to correlate the phenolic compound content with each individual antioxidant test score because each group of molecules in an extract is responsible for a specific activity. It should also be noted that for each technique used to evaluate a given activity, there are constraints and limitations that significantly influence the results. For the correlation results, at *p* = 0.05, a significant correlation is recorded between the phenolic compound contents of the extracts of the three varieties studied and the scores of the inhibition and/or reduction percentages of these same extracts. Similarly, the flavonoid content of the extracts is significantly correlated with the overall scores of the activity percentages.

### 3.5. Development of Extract-Enriched Soap Formulations

Building upon the previously demonstrated significant antioxidant activity of *O. ficus*-*indica* root extracts, this section investigates their practical application in the formulation of handmade soaps. The objective is to evaluate whether the antioxidant properties observed in vitro, along with color appearance attributes, can be effectively incorporated into cosmetic products. This endeavor aims to enhance the value of agricultural biowaste and promote the development of natural and sustainable personal care solutions. For this each variety was incorporated at three different concentrations, labeled as Soap 1: 3.33 mg/g (33.3 mg extract per 10 g soap), Soap 2: 5.00 mg/g (50 mg extract per 10 g soap), and Soap 3: 10.00 mg/g (100 mg extract per 10 g soap). A soap without any extract served as the control.

#### 3.5.1. Colorimetric Evaluation (CIELab)

**Figure 5:**
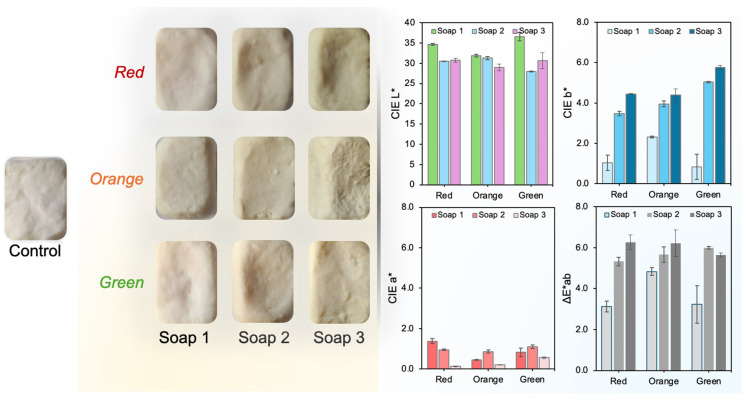
CIELab color parameters of soap formulations incorporating *O. ficus*-*indica* extracts from red, orange, and green varieties at three different concentrations.

Given the importance of the visual appearance of cosmetic products, a colorimetric analysis of the soap formulations containing *O. ficus-indica* root extracts (red, orange, and green varieties) was performed using the CIELab system (Figure 5). The reported color parameters correspond to the differences (Δ*L*, Δa*, and Δb*) relative to the control soap, which contained no added extract. This approach highlights the extent of color modification induced by the incorporation of each extract at the three tested concentrations.

As depicted in Figure 5, the lightness parameter (L*) generally decreased in all soap samples containing extracts compared to the control, indicating a darkening effect. The green variety showed the highest L* value at the lowest concentration (Soap 1: 36.59), while the orange variety had the lowest lightness at the highest concentration (Soap 3: 28.98), suggesting that both the type and concentration of extract significantly influence brightness. Regarding the a* parameter, which represents the red–green axis, a decrease was observed with increasing extract concentration for all varieties. This indicates a reduction in reddish tones in more concentrated formulations, especially for the red variety, which dropped from 1.38 (Soap 1) to 0.13 (Soap 3). The b* parameter, related to the yellow–blue axis, increased progressively with extract concentration, indicating an intensification of yellow hues—particularly evident in the orange and green soaps, which reached b* values of 4.39 and 5.77, respectively, in Soap 3. The total color difference (ΔE*ab) compared to the control soap increased with extract concentration across all samples. The highest ΔE*ab was observed in green Soap 3 (5.64), followed closely by the orange (6.21) and red (6.27) Soap 3 formulations. These values confirm that the chromatic variations were perceptible and directly proportional to extract load. Overall, these findings demonstrate that *O. ficus*-*indica* extracts significantly affect the final appearance of the soap formulations. The extract variety and its concentration can be strategically selected to modulate the visual characteristics of the product, enhancing its natural appeal and market value.

#### 3.5.2. Antioxidant Activity

**Figure 6:**
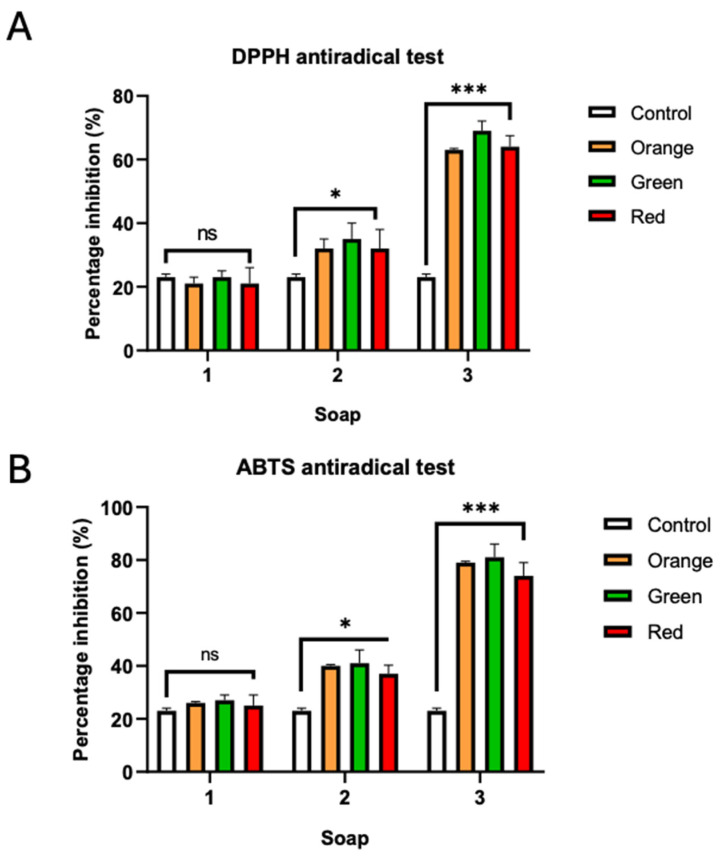
Anti-radical activity of soaps enriched with the *O. ficus-indica* root phenolic extracts from three varieties (orange, green, and red). (**A**): DPPH antiradical test; (**B**): ABTS antiradical test. ns: non-significant difference *p* > 0.05; *: significant difference at *p* < 0.05; ***: significant difference at *p* < 0.001.

Enrichment of the neutral soap base with root extracts from the three *O. ficus-indica* varieties (green, orange, and red) led to a significant enhancement of its antioxidant properties, as determined by both the DPPH and ABTS radical scavenging assays. This demonstrates a successful translation of the extracts’ antioxidant potential into a functional cosmetic formulation. Across all concentrations tested (Soap 1, Soap 2, and Soap 3), the enriched soaps consistently outperformed the control soap (lacking extract), exhibiting a clear dose-dependent increase in percentage radical inhibition (Figure 6).

Consistent with the antioxidant assessments of the root extracts themselves, where the green variety displayed potent radical scavenging activity, the soap incorporating the green root extract consistently demonstrated the highest antioxidant capacity among the soap formulations. At the highest incorporation level (Soap 3 = 10 µg/mg), this green-extract soap achieved substantial DPPH inhibition (approximately 65-70%) and particularly robust ABTS inhibition (around 90%). This suggests that the high phenolic and flavonoid content and the presence of key bioactive compounds contribute effectively to the antioxidant performance even when formulated into the soap matrix. Other studies on botanical-enriched soaps, such as those by Paputungan et al. [74], reporting 76.76% DPPH inhibition for Eucheuma spinosum soap, and Okoh et al. [75], which used neem metabolite soaps and achieved up to 85% DPPH scavenging, support the significant antioxidant levels obtainable through such natural enrichments.

The ABTS assay consistently produced higher percentage inhibition values for the enriched soaps compared to the DPPH assay (Figure 6). This differential response reflects earlier observations in this study regarding the root extracts, which were significantly more effective against the ABTS•^+^ radical (showing ABTS IC_50_ values of 29.38 ± 1.21 µg/mL for the green variety, 31.01 ± 1.13 µg/mL for the orange variety, and 35.12 ± 0.95 µg/mL for the red variety) compared to the DPPH• radical (where the green variety extract had an IC_50_ of 695.70 ± 59.30 µg/mL). Soaps formulated with these extracts at their highest concentration (Soap 3) demonstrated ABTS inhibition of approximately 90% for the green variety, around 80% for the orange variety, and about 70–75% for the red variety. The ABTS assay, primarily operating through an electron transfer (ET) mechanism, is often more sensitive to a wider range of antioxidants [76]. The pronounced activity in the ABTS assay for both the root extracts and the derived soaps suggests that the antioxidant constituents of these *O. ficus*-*indica* roots are particularly efficient ET-based scavengers (see antioxidant sub-section). This ET mechanism appears to be well-expressed within the soap matrix, possibly due to better interaction with the less sterically encumbered ABTS•^+^ radical [77]. A stable aspect highlighted by these soap formulation results is the effective retention of the root extracts’ antioxidant activity. The percentage inhibition values achieved by the enriched soaps, especially the green variety at its highest concentration, are highly encouraging, indicating minimal loss of activity during the soap-making process. The controlled melting temperature used for soap preparation (approximately 65–70 °C) seems to have been sufficiently mild to preserve the integrity of many of the bioactive phenolics detailed earlier; this heat stability is reported elsewhere for flavonoids [78]. This successful transfer of functionality from raw biowaste to a final cosmetic product is a key outcome of this valorization effort and aligns with the objective of developing sustainable cosmetic applications for these *O. ficus*-*indica* root residues. The predominant electron transfer (ET) activity demonstrated by these soaps in the ABTS assay is particularly noteworthy; such ET-based radical scavenging, strongly exhibited by the *Opuntia* root extracts themselves, suggests a capacity for the efficient neutralization of a broad range of skin-damaging free radicals [79], a potentially crucial attribute for topical soap formulations that might be more comprehensively comprehended by the ABTS method compared to the DPPH assay, which often involves hydrogen atom transfer mechanisms [80].

## 4. Conclusions

This study highlights the unexploited potential of *O. ficus*-*indica* root residues—traditionally considered agricultural waste—as a rich source of bioactive phenolic compounds with significant antioxidant properties. Among the three varieties evaluated, the green and red roots showed notably higher concentrations of phenolics and flavonoids, which were correlated with their superior performance in multiple in vitro antioxidant assays, including over 90% hydroxyl radical inhibition and effective ABTS•^+^ scavenging. Advanced phytochemical analysis revealed 26 distinct phenolic compounds (including piscidic acid, epicatechin-3-*O*-gallate, and isovitexin), many of which contribute to the extracts’ functional properties. Importantly, these bioactive-rich extracts were successfully translated into functional cosmetic formulations through their incorporation into handmade soaps. The enriched soaps retained the antioxidant efficacy of the original extracts, as demonstrated by high radical scavenging activities, particularly in the ABTS assay, where the green-variety soap reached up to ~90% inhibition. This indicates a minimal loss of antioxidant function during processing and confirms the heat stability of key phenolic constituents. Furthermore, CIELab colorimetric analysis demonstrated a clear, dose-dependent modulation of the soaps’ visual attributes—especially in terms of brightness and hue—showing that the extract variety and concentration can be strategically leveraged to enhance both the functionality and aesthetic appeal of the final product. This dual contribution—biological activity and visual enhancement—adds consumer value and aligns with market trends favoring natural, eco-conscious personal care items. Overall, this work contributes to the principles of a circular bioeconomy by valorizing underused plant residues and supporting sustainable innovation, which is particularly relevant in semi-arid and resource-constrained regions. Future efforts should explore the use of green solvents, formulation compatibility, long-term stability, and broader safety validation—critical steps toward scalable and sustainable bio-based applications.

## Figures and Tables

**Table 1:** Phenolic and flavonoid yields, expressed per gram of extract and 100 g of biomass, in *O. ficus-indica* roots.

Varieties	Total Phenolics		Flavonoids	
	mg/g Extract	mg/100 g pr	mg/g Extract	mg/100 g pr
**Green**	147.82 ± 10.33 ^a^	2920.19 ± 42.21 ^a^	125.65 ± 6.33 ^a^	2483.17 ± 25.75 ^b^
**Orange**	100.83 ± 6.11 ^c^	1392.63 ± 57.95 ^b^	82.33 ± 7.18 ^c^	1141.96 ± 33.3 ^c^
**Red**	120.29 ± 3.51 ^b^	2944.56 ± 6.31 ^a^	105.60 ± 3.88 ^b^	2591.18 ± 5.29 ^a^

The difference in letters within a column indicates a statistically significant difference according to the Tukey’s post hoc test (*p* ≤ 0.05); pr: powdered roots.

**Table 2:** Phenolic compounds identified and quantified in the hydromethanolic extracts of the roots of three varieties of *O. ficus-indica* from north Algeria, expressed per mg of extract.

Peak	RT	Tentative Identification			Green Variety		Orange Variety		Red Variety		Ref.
		Compound Name	[M-H]	MS/MS	Peak%	Conc. µg/mg	Peak%	Conc. µg/mg	Peak%	Conc. µg/mg	
1	0.83	*L*-malic acid	133	115, 87	3.124	4.62 ^a^	2.726	2.75 ^c^	4.062	4.89 ^a^	[13]
2	1.01	Citric acid	191	111, 173	3.009	4.45 ^a^	2.772	2.80 ^c^	4.123	4.96 ^a^	[13]
3	3.23	Piscidic acid	255	165,193, 179, 149	1.240	1.83 ^a^	0.943	0.95 ^c^	1.586	1.91 ^a^	[14]
4	8.41	Eucomic acid	239	179, 149	1.595	2.36 ^a^	1.699	1.71 ^c^	1.647	1.98 ^b^	[14]
5	10.15	Myricetin-*C*-hexoside	479	359	0.770	1.09 ^c^	ND	ND	2.148	2.57 ^a^	[15]
6	11.11	Carthamidin-5-*O*-glucosyle-rutinoside	757	595	1.504	2.22 ^a^	1.666	1.68 ^c^	1.664	2.00 ^b^	[16]
7	11.30	Aromadendrin-6-*C*-glucopyranosyl-7-*O*- apiofuranosyl-2-*O*-glucopyranoside	743	623, 341	1.141	1.60 ^b^	0.857	0.86 ^c^	1.363	1.64 ^a^	[17]
8	11.71	6″-*O*-sinapoyl gardoside	535	329, 205, 367	1.202	1.78 ^a^	ND	ND	1.185	1.43 ^c^	[18]
9	13.93	Sinapoyl spinacetin	569	551, 345	0.989	1.46 ^b^	0.845	0.85 ^c^	1.615	1.94 ^a^	[19]
10	14.23	Ferulic acid guaiacylglyceryl derivative	613	569, 417, 193	1.245	1.84 ^a^	0.894	0.90 ^c^	1.128	1.36 ^b^	[19]
11	14.93	Epicatechin-3-*O*-gallate	441	289	1.208	1.79 ^a^	0.773	0.78 ^c^	1.374	1.65 ^b^	[20]
12	15.93	Kaempferol-*O*-diglucoside	611	449, 287	1.197	1.77 ^a^	1.114	1.12 ^c^	1.499	1.80 ^a^	[21]
13	16.33	6-Hydroxykaempferol triglucoside/galactoside	787	625, 301, 463	1.435	2.12 ^a^	0.943	0.95 ^c^	1.403	1.69 ^b^	[16]
14	16.60	Tetragalloyle-glucose	787	635, 743, 495, 331	1.455	2.15 ^a^	0.791	0.80 ^c^	1.411	1.70 ^b^	[22,23]
15	17.65	Poricoic acid A	497	423, 453, 479	1.065	1.57 ^b^	1.114	1.12 ^c^	1.499	1.80 ^a^	[24]
16	18.93	Kaempferol-3-*O*-hexose-deoxyhexose	593	284, 285	0.863	1.28 ^a^	0.905	0.91 ^c^	1.139	1.37 ^a^	[25]
17	20.32	Phloretin-2′-xyloside	567	273	0.863	1.28 ^b^	0.982	0.99 ^c^	1.493	1.80 ^a^	[26,27]
18	21.89	Yunnaneic acid	597	579, 312, 355, 295	1.133	1.68 ^b^	1.183	1.19 ^c^	1.517	1.83 ^a^	[28]
19	22.08	Procyanidin B_1_	577	559, 425, 289	1.315	1.94 ^b^	1.253	1.26 ^c^	1.666	2.00 ^a^	[29,30]
20	24.43	Apigenin-6-*C*-glucoside (Isovitexine)	431	413, 341, 311, 269	3.086	4.56 ^a^	2.048	2.06 ^c^	3.714	4.47 ^a^	[31]
21	28.02	Quercetin-3-*O*-(6″-acétyle) hexoside	505	463, 301	1.178	1.74 ^a^	1.145	1.15 ^c^	1.349	1.62 ^b^	[32,33]
22	30.97	Tricin	329	229, 211, 183	1.313	1.94 ^a^	1.055	1.06 ^c^	1.673	2.01 ^a^	[34]
23	31.08	Isovitexin-3-hydroxy-3- methylglutaroyl	575	507, 431, 513, 473	1.164	1.72 ^c^	1.926	1.94 ^b^	1.788	2.15 ^a^	[35]
24	31.30	Trihydroxy-octadecadienoic acid	327	309, 291, 239, 195	1.335	1.97 ^a^	1.709	1.72 ^c^	1.599	1.92 ^b^	[31]
25	31.70	Placodiolic acid	375	347	1.296	1.92 ^b^	1.957	1.97 ^a^	1.081	1.30 ^c^	[36]
26	32.65	Guaiacyl-(t8-*O*-4)-guaiacyl-hexoside	537	375, 327, 179, 165	ND	ND	1.272	1.28 ^a^	ND	ND	[13]

Each value represents the mean of six aliquots from three extracts of each sample (standard deviation lower than 5%, in all cases). For all identified compounds, the SIRIUS confidence score varies between 95% and 100%, 100% being the highest score. Abbreviations: ND, not detected. Tukey’s HSD test—difference in the letter in a line indicates a significant difference at *p* ≤ 0.05 between the varieties.

**Table 3:** Antioxidant activity of *O. ficus-indica* root hydromethanolic extracts. IC_50_ or EC_50_ values given in µg/mL.

Compounds	DPPH•	ABTS•^+^	•OH	NO•	RP Fe^3+^
Green	695.70 ± 59.30 ^a^	29.38 ± 1.21 ^a^	125.33 ± 2.66 ^a^	123.82 ± 4.66 ^a^	63.77 ± 2.33 ^a^
Orange	830.37 ± 72.33 ^b^	31.01 ± 1.13 ^b^	159.22 ± 1.95 ^b^	189.30 ± 3.33 ^b^	67.99 ± 1.77 ^a^
Red	852.29 ± 28.23 ^b^	35.12 ± 0.95 ^c^	163.45 ± 5.55 ^b^	182.99 ± 4.56 ^b^	66.87 ± 5.23 ^a^
Trolox	-	6.70 ± 0.25	-	-	-
Ascorbate	5.87 ± 0.26	-	35.67 ± 0.33	32.34 ± 0.66	9.55 ± 0.25

The difference in letters in a column indicates a statistically significant difference according to the Tukey’s post hoc test (*p* ≤ 0.05).

## Data Availability

Data is contained within the article.
